# The evolution of age-specific smoking cessation rates in the United States from 2009 to 2017: a Kalman filter based approach

**DOI:** 10.1186/s12889-023-16986-w

**Published:** 2023-10-24

**Authors:** Thuy T. T. Le, Kenneth E. Warner, David Mendez

**Affiliations:** Department of Health Management and Policy, University of Michigan School of Public Health, Ann Arbor, MI 48109 USA

**Keywords:** Kalman filter, Age-group-specific cessation rates, United States, Dynamic mathematical model, Smoking prevalence

## Abstract

**Background:**

Tracking the US smoking cessation rate over time is of great interest to tobacco control researchers and policymakers since smoking cessation behaviors have a major effect on the public’s health. Recent studies have employed dynamic models to estimate the US cessation rate through observed smoking prevalence. However, none of those studies has provided annual estimates of the cessation rate by age group. Hence, the primary objective of this study is to estimate annual smoking cessation rates specific to different age groups in the US from 2009 to 2017.

**Methods:**

We employed a Kalman filter approach to investigate the annual evolution of age-group-specific cessation rates, unknown parameters of a mathematical model of smoking prevalence, during the 2009–2017 period using data from the 2009–2018 National Health Interview Surveys. We focused on cessation rates in the 25–44, 45–64 and 65 + age groups.

**Results:**

The findings show that cessation rates followed a consistent u-shaped curve over time with respect to age (i.e., higher among the 25–44 and 65 + age groups, and lower among 45-64-year-olds). Over the course of the study, the cessation rates in the 25–44 and 65 + age groups remained nearly unchanged around 4.5% and 5.6%, respectively. However, the rate in the 45–64 age group exhibited a substantial increase of 70%, from 2.5% to 2009 to 4.2% in 2017. The estimated cessation rates in all three age groups tended to converge to the weighted average cessation rate over time.

**Conclusions:**

The Kalman filter approach offers a real-time estimation of cessation rates that can be helpful for monitoring smoking cessation behavior.

**Supplementary Information:**

The online version contains supplementary material available at 10.1186/s12889-023-16986-w.

## Introduction

Adult smoking prevalence in the United States (US) decreased markedly from 2009 (20.6%) to 2018 (13.7%), according to the National Health Interview Survey (NHIS) [1]. This decreasing trend reflects desirable changes in smoking initiation and cessation rates as the result of the combined efforts of past and current tobacco control measures. While the effect of smoking initiation on mortality cannot be observed for several decades, changes in the smoking cessation rate have a near-term impact on the public’s health. Thus, monitoring fluctuations in the smoking cessation rate is essential for gaining insights into the changing tobacco use landscape.

Previous studies have estimated the US smoking cessation rate using different surveys and approaches [1–5]. In 1998, Mendez et al. used a discrete population dynamics model and NHIS prevalence data to estimate the smoking cessation rate (net of relapses) by age group [2]. Based on 1970–1993 NHIS data, the authors reported that the smoking cessation rate over both 1970–1980 and 1981–1993 increased with age. In 2022, Mendez and colleagues [1] employed another model in conjunction with NHIS smoking prevalence data to quantify the average smoking cessation rate, again net of relapses, for each 6-year period from 1990 to 2019. The authors found that the smoking cessation rate increased monotonically over the entire period (2.4% during 1990–1995, 3.4% during 1997–2001, 3.5% during 2002–2007, 4.2% during 2008–2013 and 5.4% during 2014–2019). In addition, they also reported an almost identical upward trend in the smoking cessation rate when using the 2002–2019 National Survey on Drug Use and Health (NSDUH) data.

In a 2012 article, Zhu et al. [4] analyzed NHIS data and found no upward trend in the estimated annual smoking cessation rate in the 1991–2010 period. Similarly, Zhuang et al. [5] reported no significant change in the cessation rate over time in both the 1990–2010 NHIS and 1991–2011 Tobacco Use Supplement to the Current Population Survey (TUS-CPS) data. The annual cessation rate in Zhu and Zhuang’s studies [4, 5] was defined as the percentage of smokers (having smoked at least 100 cigarettes in their lifetime) who quit smoking for at least 3 months in the past year. More recently, analyses based on the 2018–2019 TUS-CPS [6], 2015 and 2017 NHIS [7, 8] data show that the prevalence of recent successful cessation (quitting smoking for 6 months or longer within the past year at the time of the survey interview) generally decreased as age increased. None of these studies evaluated relapse, known to occur in a significant proportion of people even after having quit for six months to two years [9]. In the present work, we focused on quantifying the cessation rate as the proportion of smokers who quit each year, net of relapses (i.e., the annual net cessation rate), as in [1–3].

Kalman filters have the ability to generate efficient estimates of hidden states of a dynamic system based on noisy and indirect measurements from single or multiple data sources [10]. Furthermore, this technique can produce these estimates in real-time, allowing for the prompt generation of new estimates as soon as new observations become available. Kalman filters for parameter and/or state estimation have been used extensively in engineering applications [11–13] and other fields [14–16]. Attempts have also been made to employ these techniques to study infectious diseases [17–19]. However, no study to date has applied Kalman filters to address research questions in tobacco regulatory science where real-time surveillance of smoking behaviors is of importance and multiple data sources of the same information are available. The recent study by Mendez et al. [1] estimated smoking cessation rates based on 1990–2018 NHIS data. However, that analysis produced the average smoking rate for each 6-year period for the entire US population (not by age groups). Tracking annual cessation rates across different age groups would be beneficial in understanding the evolution of smoking behaviors.

In the present study, we use a Kalman filter based approach to estimate annual age-group-specific cessation rates among adults (25–44, 45–64 and 65 + years old), unknown parameters of a discrete mathematical model of smoking prevalence, over 2009–2017, using annual NHIS smoking prevalence data for 2009–2018. Since smoking prevalence among 18-24-year-old smokers depends on both the smoking initiation and cessation rates for that age group, we chose to exclude this age group from our analysis. We stopped the analysis in 2018 due to survey design changes in 2019 that rendered smoking prevalence not directly comparable before and after that year.

Thus, the aim of the current study was to investigate the evolution of age-specific smoking cessation rates in the US.

## Methods

In this section, we first introduce a discrete mathematical model of smoking prevalence utilized to quantify annual smoking cessation rates for the three specified age groups (25–44, 45–64, 65+) [2]. Then, we discuss how to apply a Kalman Filter to the proposed model to estimate the unknown cessation rates using the 2009–2018 NHIS prevalence data. Here the cessation rate refers to the annual net cessation rate defined in Mendez’s work [3, 20].

### Mathematical model

To estimate the cessation rates by age group for each year from 2009 to 2017, we employed the following dynamic mathematical model adapted from [2].

1 $$\begin{array}{c}{C}_{25,t}={\gamma }_{t }\times {P}_{25,t} \end{array}$$

2 $$\begin{array}{c}{\text{C}}_{a,t }={\text{C}}_{a-1,t-1}\times \left(1-{\mu }_{a-1,t-1}\right)\times \left(1-{\theta }_{a-1,t-1}\right),a =26,\dots ,100 \end{array}$$

3 $$\begin{array}{c}{R}_{\left[{a}_{i},{a}_{j}\right],t}=\frac{\sum\nolimits_{a={a}_{i}}^{{a}_{j}}{C}_{a,t } }{\sum\nolimits_{a= {a}_{i}}^{{a}_{j}}{P}_{a,t}},\end{array}$$

Where ${{\text{C}}_{a,t },\mu }_{a,t}, {\theta }_{a,t}$ and ${P}_{a,t}$ are, respectively, the number of current smokers, the death rate of current smokers, the smoking cessation rate and the US population at age *a* and in year $t$. ${R}_{\left[{a}_{i},{a}_{j}\right],t}$ is the smoking prevalence among ${a}_{i}-{a}_{j}$ year-olds in year $t$. For our three age groups, $\left[{a}_{i},{a}_{j}\right]\in \left\{\left[\text{25,44}\right], \left[\text{45,64}\right], \left[\text{65,100}\right]\right\}$. The parameter ${\gamma }_{t }$stands for the smoking initiation rate in year $t$. Here, we aimed at quantifying the annual net cessation rates for each of the three specified age groups. As such, the cessation rate was kept constant within each group which results in three unknown parameters to be estimated every year,

4 $$\begin{array}{c}{\theta }_{a,t }= \left\{\begin{array}{c}{\eta }_{1,t}\;a\in \left[\text{25,44}\right],\\ {\eta }_{2,t }\;a\in \left[\text{45,64}\right],\\ {\eta }_{3,t }\;a\in \left[\text{65,100}\right].\end{array}\right.\end{array}$$

A Kalman filter can be applied to estimate the unknown cessation rate ${\theta }_{a,t}$ in Expressions (1–4) by rewriting the model as follows

5 $$\begin{array}{c}{\theta }_{a,t }= {\theta }_{a,t-1 }+{w}_{a, t-1}\end{array}$$

6 $$\begin{array}{c}{C}_{25,t}={\gamma }_{t }\times {P}_{25,t} \end{array}$$

7 $$\begin{array}{c}{\text{C}}_{a,t }={\text{C}}_{a-1,t-1}\times \left(1-{\mu }_{a-1,t-1}\right)\times \left(1-{\theta }_{a-1,t-1}\right), a=26,\dots ,100 \end{array}$$

8 $$\begin{array}{c}{D}_{\left[{a}_{i},{a}_{j}\right],t}=\frac{\sum\nolimits_{a={a}_{i}}^{{a}_{j}}{C}_{a,t } }{\sum\nolimits_{a= {a}_{i}}^{{a}_{j}}{P}_{a,t}}+{v}_{\left[{a}_{i},{a}_{j}\right],t}\end{array}$$

Where ${w}_{a, t}$ is the parameter noise at age $a$ in year $t$, ${D}_{\left[{a}_{i},{a}_{j}\right],t}$ and ${v}_{\left[{a}_{i},{a}_{j}\right],t}$ are the NHIS-observed smoking prevalence and the random noise term among ${a}_{i}-{a}_{j}$ year-olds in year $t$. The random term ${v}_{\left[{a}_{i},{a}_{j}\right],t}$, the measurement error, was assumed to be Gaussian zero-mean white noise with the estimated standard error of the NHIS observed smoking prevalence as the standard deviation. The parameter noise ${w}_{a, t}$ was assumed to be normally distributed with zero mean. As the cessation rates ${\theta }_{a,t}$ are likely to be time-varying, the variance matrix of ${w}_{a, t}$ was chosen based on the “forgetting-factor” method, see Chapter 7 in [21]. Equation (5) was used to obtain a prior estimate of the cessation rate, which was then updated using new available observation(s).

The age-specific population for each year during the 2009–2018 period was extracted from the US Census Bureau [22]. The 2009 smoking prevalence by single age and the annual smoking prevalence by age group over the 2009–2018 period were taken from 2009 to 2018 NHIS [23–26]. The cessation rate in a specific year, say 2009, reflects the rate of quitting that occurs between 2009 and 2010, and its calculation requires NHIS-observed prevalence for both 2009 and 2010. As such, we need 2009–2018 NHIS data for estimating 2009–2017 cessation rates. The adult smoking initiation rate ${\gamma }_{t }$was set equal to the 25-year-old smoking prevalence in year $t$ estimated from the NHIS data. With this choice of ${\gamma }_{t }$, we captured all current smokers who initiated before age 26, but ignored the very few smokers who initiated after age 25 [27]. The effect of this omission on our results is negligible, due to the small number of individuals who start smoking after the age of 25. We used the Cancer Intervention and Surveillance Modeling Network (CISNET) age-specific mortality rates of current smokers for each year during the considered period [28, 29]. Every year the number of current smokers aged 25 is a product of the corresponding initiation rate and the 25-year-old population. The number of smokers aged 26 and over was computed as the number of smokers who were a year younger in the previous year, and who survived and continued smoking in the current year. Finally, the smoking prevalence in each of the three age groups was obtained by dividing total current smokers by the total adult population within that age group.

The weighted average cessation rate for the population aged 25 and over in year $t$ was calculated as the sum of the products of the number of current smokers in each age group at the beginning of year $t$ and the corresponding cessation rate divided by the total current smokers aged 25 and over at the beginning of that year.  

### Kalman filter for parameter estimation

**Fig. 1:**
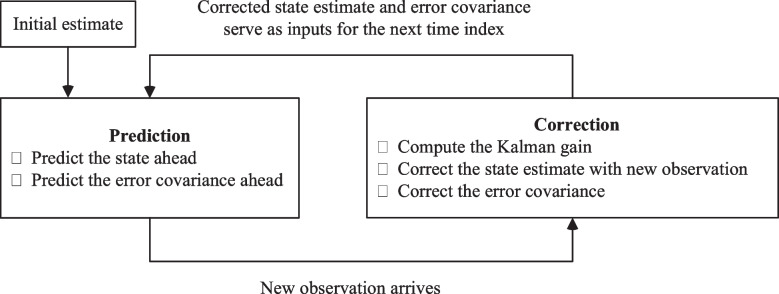
Diagram of a Kalman filtering technique

Kalman filter based methods utilize recursive Bayesian updates to estimate an unknown variable in a dynamical system while accounting for the uncertainty of observations [10]. These approaches generate a prior estimate of the unknown variable based on all the previously available observations and the given system dynamics, and then refine this estimate with new observation(s) to obtain a posterior estimate as illustrated in Fig. 1.

To maintain a desirable balance between accuracy and computational tractability of a filtering method, we utilized the Central Difference Kalman filter (CDKF) [30, 31] to estimate the annual smoking cessation rates ${\theta }_{a,t }$, using the mathematical model in Eqs. (5, 6, 7 and 8) and the annually noisy NHIS-observed smoking prevalence. In this work, we assumed that all random variables are Gaussian [21]. The detailed algorithm of the CDKF can be found in [21, 31]. The initiation rate, the NHIS-observed smoking prevalence and its estimated standard error for each age group are shown in Table A1 in the Additional file 1.

All methods were performed in accordance with the relevant guidelines and regulations.

## Results

The estimated cessation rates with their standard deviations for the three age groups, as well as the weighted average rate with its standard deviation from 2009 to 2017, are shown in Table 1. Overall, the cessation rates in the 25–44 and 65 + age groups remained nearly unchanged at approximately 4.5% and 5.6%, respectively, during the studied period. Meanwhile, the rate in the middle age group increased substantially from 2.5% to 2009 to 4.2% in 2017. The cessation rates in the youngest and oldest age groups are initially, in 2010, more than double the rate in the middle age group. However, this gap among these age groups had narrowed by 2017 with the rate among 45-64-year-olds approaching the rate in the 25–44 age group. Lastly, the weighted average rate, which was computed by averaging the estimated rates of these age groups weighted by their age-group-specific numbers of current smokers, was stable at around 3.7% until 2013 before increasing to 4.5% by the end of the 2009–2017 period.

**Table 1:** Estimated age-group-specific cessation rates with standard deviations

Year	Cessation Rate (Standard Deviation)			
	Age Groups			Weighted Average
	25–44	45–64	65+	
2009	4.98% (0.80%)	2.46% (0.80%)	4.86% (0.84%)	3.89% (0.52%)
2010	4.76% (0.77%)	2.07% (0.79%)	5.50% (0.84%)	3.66% (0.51%)
2011	4.46% (0.76%)	2.44% (0.77%)	5.63% (0.85%)	3.69% (0.50%)
2012	4.44% (0.75%)	2.40% (0.76%)	5.69% (0.86%)	3.67% (0.48%)
2013	4.13% (0.74%)	2.95% (0.75%)	5.81% (0.87%)	3.79% (0.48%)
2014	4.44% (0.73%)	3.69% (0.74%)	5.87% (0.88%)	4.27% (0.47%)
2015	4.46% (0.73%)	3.73% (0.73%)	5.68% (0.89%)	4.27% (0.47%)
2016	4.69% (0.73%)	4.08% (0.73%)	5.62% (0.90%)	4.53% (0.47%)
2017	4.50% (0.73%)	4.17% (0.73%)	5.49% (0.91%)	4.48% (0.47%)

**Fig. 2:**
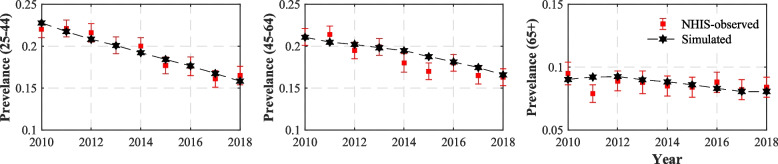
Simulated and NHIS-observed prevalence with 95% error bars for all age groups from 2010 through 2018

Figure 2 displays the simulated smoking prevalence for each age group, which was obtained through the model simulations using the estimated cessation rates. The NHIS-reported smoking prevalence with its 95% CIs is also included in the figure for comparison. In all age groups, the simulated smoking prevalence aligns closely with the NHIS-observed prevalence of adult current cigarette smoking (most within the 95% confidence intervals of the NHIS-observed smoking prevalence). This lends confidence in our estimated smoking cessation rates (Readers can find all the numeric values used to produce Fig. 2 in Table 1A in the Additional file 1).

**Fig. 3:**
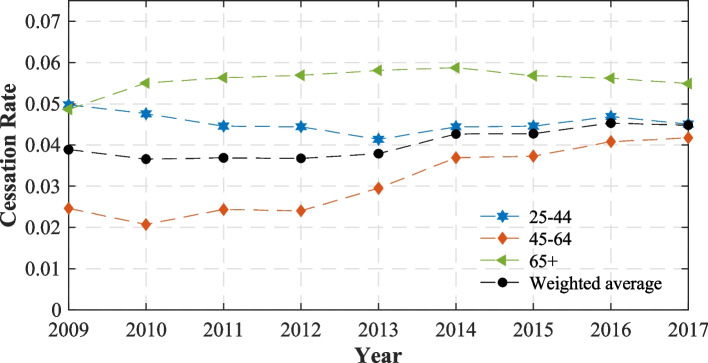
Smoking cessation rates of three age groups relative to the weighted average rate

Figure 3 displays age-group-specific cessation rates relative to the average rate, weighted by the proportion of smokers in each group. During the period, we found that the magnitude of smoking cessation rates (net of relapses) is ordered in a consistent U-shape with respect to age. The cessation rate was highest among the 65 + age group, while the lowest rate was among 45-64-year-olds. However, the rates of these groups tended to converge to the weighted average value by the end of the period.

## Discussion

We have demonstrated the use of a Kalman filter approach to estimate annual age-group-specific smoking cessation rates, unknown parameters of a mathematical model of smoking prevalence, from 2009 to 2017. Our study represents the first of its kind to employ this methodology in tobacco control. This approach can be used to develop a monitoring tool that provides tobacco researchers and regulators with timely information about changes in tobacco use trends.

Our findings indicate a u-shaped curve with respect to age: the rate is higher among the 25–44 and 65 + age groups than among 45-64-year-olds. This is consistent with [32], where the results show that, for the period 2001–2010, the cessation rate achieved the highest value of 7.1% among 25-44-year-olds, decreasing to 4.7% for the 45–64 age group, and rising again to 5.3% for the older (65+) group. However, our results do not match with other studies [7, 8] in which the authors found that the cessation rate decreased monotonically as age increased. The inconsistent findings could be attributed to the varying definitions of cessation rate used across different studies. For instance, the authors in [7, 8] defined this rate as the ratio of former smokers who stopped smoking for at least 6 months during the past year to the number of current smokers who smoked for more than 2 years and former smokers who stopped smoking during the past year. With this definition, as discussed in [7], the estimated recent cessation rate is likely to overestimate the net quitting rate due to relapse by some former smokers. Meanwhile, in our present study, we estimated annual cessation rates net of relapse as in [1, 3].

When comparing the cessation rate of each age group to the weighted average rate, we observed that all the estimated rates tended to start converging to the average since 2013. The weighted average cessation rate increase observed between 2014 and 2017 can be attributed to several factors. In particular, the cessation rates in the middle age group rose considerably during this period, while the rates in the other age groups were reasonably unchanged, although they remained consistently the highest age-group-specific rates. Notably, the rate among 45-64-year-olds rose by 70% during the studied period, increasing from 2.5% to 2009 to 4.2% in 2017.

One might interpret the u-shaped cessation rates by age groups as indicating challenges or less interest in quitting smoking among 45-64-year-old smokers. Their lower cessation rates might suggest the need for directing more attention and resources towards encouraging smoking cessation within this age group. However, given that the group’s cessation rate exhibited a rapid increase and nearly caught up with the rates in the other age groups, focusing on these other age groups, where the cessation rates remained quite stable from 2009 to 2017, might warrant equal attention.

Our study estimated lower average smoking cessation rates during the 2009–2017 period compared with those reported in [1]. Our estimated rate averaged approximately 4.4% over 2014–2017, whereas Mendez’s analysis [1] resulted in an average rate of 5.4% for 2014–2019. It is important to note that the weighted average cessation rate reported in this study and the average rate in [1] were estimated in different populations. Specifically, we calculated the average cessation rate for smokers aged 25 or older, while Mendez and colleagues evaluated cessation by the entire adult smoking population, including the 18–24 age group. The inclusion of this younger group, which has been shown to have a high cessation rate [7, 8], may have a significant impact on the reported average cessation rate in [1]. As well, Mendez and colleagues [1] estimated cessation to 2019.

Our estimated age-group-specific cessation rates can be used to develop projections of tobacco use trends going forward for various purposes. To illustrate, we can employ these estimates in our simulation model, along with the 2017 input data, to project US smoking prevalence to the year 2030. This permits us to assess the likelihood that the nation will achieve CDC’s Healthy People goal of 5% for US adult smoking prevalence in that year (revised to 6.1% in 2021 due to the 2019 NHIS redesign) [33]. This application of the cessation rate estimates in our model projects smoking prevalence to be 8.8% (95% CI: 7.9 − 9.6%) in 2030. This is close to the 2022 Mendez et al. [1] prediction that smoking prevalence in 2030 would be around 8.3% (95% CI: 4.6 − 16.8%). However, neither of these projections allowed for further decreases in the smoking initiation rate and increases in cessation rates, both likely given recent trends in these important variables. Had we incorporated continuation of those trends, our analysis suggests that, while the Healthy People goal is not likely to be achieved, actual prevalence could get quite close to the CDC target.

This work has some limitations. First, the initiation rate was chosen based on the smoking prevalence of 25-year-olds, meaning that the contribution of people who started smoking after the age of 25 was not considered. We believe this has minimal impact on our results since very few smokers initiate after age 25 [27]. Secondly, we assumed that the dynamics of smoking prevalence has zero error term, i.e., no modeling misspecification (see Eqs. (1 and 2)), which is unlikely to hold for many models in practice. However, this model has been proven to track smoking prevalence very accurately in previous studies [34]. Moreover, as shown in this study, the simulated smoking prevalence captures well the observed NHIS prevalence, which gives us confidence in our estimates. Lastly, our analysis was stopped in 2018, five years ago. While it would have been desirable to include 2019–2022 in our analysis, the survey design changes in 2019 make a direct comparison of smoking prevalence before and after that year infeasible. Consequently, we could not extend the analysis to incorporate the 2019–2022 NHIS data.

## Conclusions

Our findings show how smoking cessation rates changed absolutely and relatively across different age groups from 2009 to 2017. As the first study to employ a Kalman filter technique to estimate cessation rates by age groups - unknown model parameters - our analysis demonstrates the potential application of this approach to other issues in tobacco control. This method offers researchers and tobacco regulators a novel mechanism for surveillance of tobacco use trends.

### Supplementary Information

**Additional file 1: Table 1A. **The NHIS-observed smoking prevalence together with its standard errors and the initiation rate γ_t. SE: standard error.

## Data Availability

All data generated or analyzed during this study are included in this published article and its supplementary information file.
