# Supplementary material for: The evolution of age-specific smoking cessation rates in the United States from 2009 to 2017: a Kalman filter based approach
Source: BMC Public Health. 2023 Oct 24;23:2076. doi: 10.1186/s12889-023-16986-w (PMC10594685; doi:10.1186/s12889-023-16986-w)
Supplement: Supplementary file 1 — Additional file 1: Table 1A. The NHIS-observed smoking prevalence together with its standard errors and the initiation rate γ_t. SE: standard error. [file 12889_2023_16986_MOESM1_ESM.docx]

| **Year** | **Prevalence**  **(25-44)** | **SE**  **(25-44)** | **Prevalence**  **(45-64)** | **SE**  **(45-64)** | **Prevalence (65+)** | **SE (65+)** | **Initiation Rate (**$\gamma_{t})$ |
| --- | --- | --- | --- | --- | --- | --- | --- |
| 2009 | 24.00% | 0.56% | 21.90% | 0.66% | 9.50% | 0.51% | 28.80% |
| 2010 | 22.00% | 0.51% | 21.10% | 0.51% | 9.50% | 0.46% | 28.00% |
| 2011 | 22.10% | 0.51% | 21.40% | 0.51% | 7.90% | 0.36% | 21.70% |
| 2012 | 21.60% | 0.56% | 19.50% | 0.51% | 8.90% | 0.41% | 25.30% |
| 2013 | 20.10% | 0.51% | 19.90% | 0.51% | 8.80% | 0.46% | 22.80% |
| 2014 | 20.00% | 0.51% | 18.00% | 0.56% | 8.50% | 0.41% | 22.40% |
| 2015 | 17.70% | 0.51% | 17.00% | 0.51% | 8.40% | 0.41% | 18.60% |
| 2016 | 17.60% | 0.56% | 18.00% | 0.51% | 8.80% | 0.41% | 18.60% |
| 2017 | 16.10% | 0.51% | 16.50% | 0.51% | 8.20% | 0.41% | 15.43% |
| 2018 | 16.50% | 0.56% | 16.30% | 0.51% | 8.40% | 0.41% | 11.90% |

**ADDITIONAL FILE 1 TO “THE EVOLUTION OF AGE-SPECIFIC SMOKING CESSATION RATES IN THE UNITED STATES FROM 2009 TO 2017: A KALMAN FILTER BASED APPROACH”**

Thuy T.T. Le PhD, Kenneth E. Warner PhD, David Méndez PhD

Department of Health Management and Policy, University of Michigan School of Public Health, Ann Arbor, MI 48109, USA

**Table 1A**: The NHIS-observed smoking prevalence together with its standard errors and the initiation rate $\gamma_{t}$. SE: standard error.
